# Effect of individualized communication skills training on physicians’ discussion of clinical trials in oncology: results from a randomized controlled trial

**DOI:** 10.1186/s12885-017-3238-0

**Published:** 2017-04-13

**Authors:** Alexander Wuensch, Tanja Goelz, Gabriele Ihorst, Darcey D. Terris, Hartmut Bertz, Juergen Bengel, Michael Wirsching, Kurt Fritzsche

**Affiliations:** 1 Center for Mental Health, Department of Psychosomatic Medicine and Psychotherapy, Medical Center - University of Freiburg, Faculty of Medicine, Hauptstr. 8, D-79104 Freiburg, Germany; 2 Department of Internal Medicine I (Hematology and Oncology), Medical Center – University of Freiburg, Faculty of Medicine, Hugstetterstr. 55, D-79106 Freiburg, Germany; 3 Center for Pediatrics, Department of General Pediatrics, Adolescent Medicine and Neonatology, Medical Center – University of Freiburg, Faculty of Medicine, Mathildenstr.1, D-79106 Freiburg, Germany; 4 Clinical Trials Unit (CTU), Medical Center – University of Freiburg, Faculty of Medicine, Elsaesser Str. 2, D-79110 Freiburg, Germany; 5 Center for Family Research, University of Georgia, 1095 College Station Rd, Athens, GA 30602 USA; 6 Institute of Psychology, Department Rehabilitation Psychology and Psychotherapy, Albert-Ludwigs-University Freiburg, Engelbergerstr. 41, D-79106 Freiburg, Germany; 7 Psychosomatic Medicine and Psychotherapy, Klinikum rechts der Isar, Technical University of Munich, Langerstrasse 3, D-81675 Munich, Germany

**Keywords:** Communication skills training, CST, Oncology, Randomized clinical trials, Informed consent

## Abstract

**Background:**

Discussing randomized clinical trials (RCTs) with cancer patients is one of the most challenging communication tasks a physician faces. Only two prior Communication Skills Trainings (CSTs) focused on RCTs in oncology have been reported. Their results demonstrated the need for further improvement. We developed and evaluated an enhanced, individually-tailored CST focused on improving physicians’ communication during discussions of RCTs.

**Methods:**

The CST focused on personal learning goals derived from video pre-assessment that were addressed in a 1.5-day group workshop and one-on-one coaching sessions. Forty physicians were recruited and randomly assigned to intervention and control groups. Video-recorded standardized consultations with actor-patients were utilized. As a primary outcome (1), training success was evaluated by blinded raters using a previously developed checklist. Change in checklist items was evaluated between pre- and post-training assessment and compared against control group results. As a secondary outcome (2), the physicians’ feeling of confidence was assessed by a questionnaire.

**Results:**

(1) Significant improvements in the intervention group were observed for the score on all items (*p* = 0.03), for the subgroup of content-specific items (*p* = 0.02), and for the global rating of communication competence (*p* = 0.04). The improvement observed for the subgroup of general communication skill items did not achieve significance (*p* = 0.20). (2) The feeling of confidence improved in nine out of ten domains.

**Conclusion:**

While the individually-tailored CST program significantly improved the physicians’ discussions of RCTs, specifically related to discussion content, what remains unknown is the influence of such programs in practice on participant recruitment rates.

The study was registered retrospectively in 2010/07/22 under DRKS-ID: DRKS00000492.

**Electronic supplementary material:**

The online version of this article (doi:10.1186/s12885-017-3238-0) contains supplementary material, which is available to authorized users.

## Background

Randomized clinical trials (RCTs) are seen as the gold standard to improve cancer care [1]. On average, only a minority of eligible patients take part in RCTs [2–5], depending on various factors, e.g., age, sex or cultural milieu [6]. This low participation rate, and the factors that influence participation, may result in bias compromising the external validity of results. RCTs do not guarantee benefit to their participants. The benefit may be experienced by current participants, but benefit depends on assignment to the treatment arm and more typically arises for future patients when new therapeutic regimens are established [5]. Under these conditions, potential RCT participants must consider the risks and burdens associated with participation against potential advantages. As a result, one of the most challenging communication tasks in oncology is to discuss RCTs with patients, with informed consent requiring an elaborated communication process before patients’ decision-making [7].

Previous studies have shown that patients often lack a basic understanding of crucial aspects of RCTs [8]. Recall of the information presented when RCTs are discussed is low [9] and many patients report false beliefs about the likely benefits and risks [10]. When discussing RCTs, patients find the possibility of random allocation to a control arm least acceptable [11]. In the face of these challenges, improving the clarity of information when RCTs are presented can support patients’ decision-making. In addition to ensuring a patient comes to a free and fully-informed decision, improved communication may also increase the likelihood that a patient will choose to participate [9, 12–14].

Accrual to clinical trials is influenced by various stakeholders and factors at different levels of the health care system. These include: i) the disclosing physician, e.g., their prior experience [2]; ii) the patient, e.g., their understanding of clinical trials [12] or perceived value of the trial [15]; iii) the set-up of the trial work, e.g., team involvement [5] or organization of trial recruitment; iv) the study protocol, e.g., benefits or burdens of study participation, including extra time required for study participation [16] and v) applicable health policy, e.g., national program support [17]. A key point in study recruitment, however, remains communication.

The communication skills of physicians working in oncology can be improved by specific training programs [17–26], with studies [27] and meta-analysis [28, 29] showing moderate effect sizes (ES = .54). For example, Brown [1, 30] developed a one-day CST, trained ten oncologists, and evaluated 90 audiotaped informed consent consultations with real patients in a pre-post design. The study’s results demonstrated significant improvements for three of 25 items related to the clinical and ethical information provided. In the work of Jenkins [12], 68 research nurses and 33 oncologists were trained to convey key information about RCTs. The training included watching eight hours of videotaped RCT consultations to trigger participants’ discussion and practice of communication techniques. The training effects were evaluated using videotaped consultations with actor-patients and a checklist in a pre-post-design. Significant improvement was observed for participants’ delivery of key information for 10 out of 25 items.

Still, few interventions have been developed to focus on the specific challenges of discussing RCTs with patients [1, 12, 30] and very few focus precisely on communication skills training (CST) [1, 12, 30]. Further, the evaluation of this prior oncology RCT-focused CST has been limited by the lack of randomized control groups [1, 12], the use of short training times [1, 12], and the inclusion of a wide range of topics in training [1, 30].

We follow CONSORT guidelines in this publication.

## Methods

### Trial design

To rigorously investigate the effects of the individualized CST developed, we utilized a randomized trial design and compared the observable communication skills of an intervention and control group to test for significant changes and differences.

### Participants

Physicians working in the field of oncology and involved in RCTs were eligible to participate. The cover fee for training was € 50. Physicians were recruited from the departments of internal medicine (specifically oncology), gynecology, and surgery at the University Hospitals Freiburg and Ulm (Germany) and two affiliated hospitals.

### Intervention

The CST utilized in our study was developed based on the prior work of Brown [1, 29] and Jenkins [12] and met most of the recommendations of a consensus report for CST in oncology [25]. Specifically, the CST included pre-assessment of participants’ communication skills, followed by a 1.5-day group workshop and one-on-one coaching sessions that covered 17 h in total.

During the pre-assessment, participants discussed oncology RCTs in sessions with trained actor-patients. The CST workshops were then held for groups of eight participants. In the workshops, there was theoretical input about communication and ethical guidelines. The groups were then further divided into subgroups (*n* = 4) for role play with actor-patients. Feedback on role play activities was provided by the trainers, participants’ peers, and the actor-patients. The one-on-one coaching sessions were held two weeks later and discussed ways of transferring acquired skills into everyday practice. Further details concerning the CST can be found in Wuensch [31].

### Outcomes

We evaluated whether the individually-tailored CST improved physicians’ communication skills when discussing oncology RCTs with actor-patients. The quality of the participants’ communication skills as a primary outcome was assessed based on the content of the discussions, general communication skills demonstrated, and overall communication competence. See Table 1. As a secondary outcome, we assessed study participants’ feeling of confidence using a questionnaire including ten items and employing a 10 cm log Visual Analogue Scale [32].

**Table 1:** Items of the COM-ON-rct-checklist

Content-specific items: Disclose information about clinical trails	
Did the participant:	
• Explore the patient’s perception of the situation?	
• Set an agenda for the discussion?	
• Maintain sequences of treatment options (first standard then trials)?^a^	
• Introduce and explain treatment options?	
• Explain the set up and process of the research project?	
• Provide an appropriate explanation of randomisation?	
• Explain the reason for randomisation?	
• Explain possible risks and side-effects?	
• Explain that unknown effects may occur?	
• Explain that participation is voluntary?	
• Close the discussion in an appropriate manner?	
General Communication Skills-related Items	
Did the participant:	
• Achieve an appropriate beginning?	
• Use appropriate language?	
• Employ adequate nonverbal communication?	
• Take pauses?	
• Show empathy to the patient?	
• Encourage the patient to ask questions?	
• Employ an adequate way to check the patient’s understanding?	
• Structure the discussion?	
Overall Evaluation Item	
• What is your overall evaluation of the participant’s communication competence?	

^a^Binary item (yes/no) not integrated in the Mixed Model analysis utilized in the evaluation

### Sample size

The required sample size was estimated based on the related work of Langewitz [33] who found large effect sizes (ES) =1.29. At the time of development of this study, this was one of the few data available on effect sizes associated with changes in communication skills involving complex information. To detect an ES = 1.0, with a power of 80% and a significance level of 5% (two sided t-test), 17 participants per group were required. Considering drop-outs, we aimed to recruit 40 physicians.

### Randomization

**Fig. 1:**
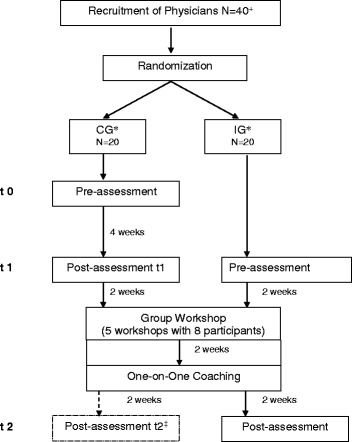
Flowchart of study design. Research design for the evaluation of the developed CST. Each assessment period included two randomized controlled trial (RCT) discussion sessions for each participant. The RCT discussion sessions utilized standardized RCT scenarios based on real-life RCTs and trained actor-patients. ^+^ An additional physician was initially recruited for the study but dropped out after the pre-assessment sessions due to scheduling difficulties. This early drop-out was not included in the analysis.* CG = Control Group, IG = Intervention Group. ^‡^ Data of post-assessment t2 of intervention group was not analyzed in this study

Participants were randomly assigned to the intervention group (IG) or a waiting-list control group in blocks of eight participants (see Fig. 1). Group assignment was based on a computer-generated randomization list prepared by the Clinical Trials Unit (Studienzentrum), University Medical Center Freiburg. The study design allowed the control group (CG) to eventually participate in the CST.

The effect of the CST was evaluated by trained raters who reviewed videotapes of the RCT discussions held between the participants and actor-patients. The raters were blinded as to participants’ assignment to the IG or CG. Initial discussion sessions, before CST, were used for the pre-assessment phase of the study. Two weeks following the CST participants’ completion of the one-on-one coaching sessions, the second set of discussion sessions were held. Two discussion sessions were rated for each participant at each time point with a developed checklist [34].

The assessment of RCT discussion sessions was standardized to minimize the influence of RCT attributes and patient characteristics. Six RCTs conducted at the University Hospital Freiburg were used as templates for the RCTs addressed during discussions, with the descriptions simplified to approximate a similar level of study complexity (supervised by HB). We included a spectrum of different types of phase III trials, testing either (a) a placebo against a new drug or (b) a standard treatment against an optimized treatment. Information about the RCTs was summarized to a level typically found in Internet descriptions (e.g. http://clinicaltrials.gov/).

The actor-patients were also trained in two specified roles. In the first role, the patient was described as an “Internet-expert” who was critical of the randomization process and would emphasize a perceived right to be included in the treatment arm. In the second role, the patient was also described as critical of RCTs and distrustful of giving up control, especially for the purpose of randomization.

## Statistical methods

The previously developed COM-ON-Checklist used by the raters [34] included 20 items with sub-groups related to the content of the RCT discussions and participants’ general communication skills, with a single item providing a global assessment of participants’ communication competency. Nineteen items, scored on a 5-point scale, were included in the main analysis. Mean scores were calculated for sub-grouped items (e.g. content-related and general communication skill items), and a Mixed Model was applied. The remaining item included on the checklist asked for a binary response (yes/no) as to whether the standardized treatment was discussed first before introducing the RCT. When evaluating the binary item for participants across the two RCT discussion scenarios, three outcomes were possible: never maintained sequence (0), maintained sequence in one of the scenarios (1), and maintained sequence in both scenarios (2). This item was analyzed separately from the previously described 19 items by applying a Chi^2^-Test.

Three raters evaluated the video-recorded RCT discussions. The raters were trained to use the checklist with 30 demonstration videos. All of the raters had a theoretical background related to the checklist and knowledge of the risks of bias in rating procedures. In an iterative process, the training of the raters was continued until a satisfactory interclass coefficient (ICC) was achieved (ICC = .70 for content-specific items, ICC = .80 for general communication skills, and ICC = .50 for the global assessment of communication competency) [34].

The analysis of the change in item scores was performed using a Mixed Model in SAS statistical software, version V9.2 for Linux (SAS Institute Inc., Cary, NC, USA). The distribution of the data was checked and, for grouped items, considered to be sufficiently close to the normal distribution to justify this procedure. Although for single items the application of the same type of model seems appropriate, the normality assumption must be considered as an approximation as the scales comprise five values (0–4), with few averaged values due to the multiple ratings included.

The change in item scores after CST participation for the IG and after four weeks without training for the CG was used as the outcome variable. Treatment effects were estimated controlling for fixed effects associated with the RCT scenarios and random effects associated with the study participants. To analyze the differences between the pre- and post-assessment, adjusting for different baseline scores, the baseline item scores were incorporated as a covariate. No alpha-adjustment was made in the model as we did not intend to demonstrate effects for specific items, but were interested in more general patterns related to item sub-groups. Hence, the *p*-values reported for individual items should be regarded as descriptive. The ES was derived from the intervention effect estimate and the estimated standard deviation of observations obtained from the random effects model. We additionally collected data from our control group who were asked to do a post-assessment t2 after the workshop in concordance with our intervention group. However, we did not analyze these data for this study.

The binary item was analyzed by performing a Chi^2^-Test comparing the sum score of two scenarios obtained after CST participation for the IG and after four weeks without training for the CG (Mantel Haenszel test for the alternative of a linear trend). The secondary outcome was calculated similarly to the main analysis, employing a Mixed Model.

## Results

### Participation flow and recruitment

Forty-one physicians were recruited (AW, TG) to participate in the study. One physician withdrew his participation after pre-assessment because of scheduling difficulties, leaving 40 participants who completed the study protocol and were included in data analysis (see also Fig. 1).

### Baseline data and numbers analyzed

The participants were mostly junior physicians (*n* = 37) and were, on average, 33 years old with five years of experience (see Table 2). Only six participants had prior communication skills training. CG participants had, previous to the current CST, discussed information about RCTs with an average of 5.6 patients (SD 5.8, median 5, range 0–20) while IG participants had previously discussed information about RCTs with an average of 10.8 patients (SD 11.9, median 8, range 0–45). The IG and CG did not differ statistically.

**Table 2:** Sample description

	Control group (CG)	Intervention group (IG)
N	20	20
Males (%)	7 (35.0%)	11 (55.0%)
Age in Years^a^	33 (5.3)	32 (4.0)
Years of Professional Experience^a^	4.5 (3.7)	4.9 (4.0)
No. of patients with whom RCT are discussed per quarter of a year^b^	5.6 (5.8)	10.8 (11.9)
Prior Communication Training (%)	4 (20.0%)	2 (10.0%)
Resident Doctors (%)	18 (90.0%)	19 (95.0%)
Specialization		
Internal Medicine (%)	8 (40.0%)	10 (50.0%)
Gynaecology (%)	7 (35.0%)	4 (20.0%)
Surgery (%)	4 (20.0%)	4 (20.0%)
Radio–oncology (%)	1 (5.0%)	2 (10.0%)

^a^Age and Years of Professional Experience are given as mean (st.dev.) in years

^b^Mean and (st.dev)

All other values are reported as n (%)

### Outcomes and estimations

Regarding the primary outcome, significant differences were observed between the IG and CG for a change in item scores across all items pooled together (*p* = 0.03), for the sub-group of content-specific items (*p* = 0.02), and for the global rating of communication competence (*p* = 0.04) (see Table 3). The observed ESs were moderate: ES = 0.58 for all items, ES = 0.57 for content-specific items, and ES = 0.52 for the global rating. Although an improvement for the sub-group of general communication skill items for the IG versus CG was observed, this difference did not achieve significance (*p* = 0.12). On an individual -item basis, four of the nine content-specific items demonstrated a significant improvement, specifically: “explain the reason for randomization” (*p* = 0.01), “explore patient’s perception” (*p* = 0.04), “define unknown effects of study” (*p* = 0.03), and “explain set-up of research project” (*p* = 0.04).

**Table 3:** Mixed models results of subgroup items. Results of subgroup-items

Item	Effect	Estimates	Standard error	Confidence interval 95%	p	Effect sizes^a^
All items	Δ IG t_2_	0.2840	0.1257	0.02947–0.5386	0.0297	0.5828
Subgroup: content specific communication skills	Δ IG t_2_	0.3066	0.1303	0.04279–0.5704	0.0239	0.5694
Subgroup: general communication skills	Δ IG t_2_	0.2154	0.1643	−0.1173 - 0.5481	0.1978	0.3632
Single item: global rating	Δ IG t_2_	0.3960	0.1824	0.02671–0.7652	0.0363	0.5204

Mixed model with baseline as covariate

^a^Derived by the formula: $d\kern0.5em \approx \kern0.5em \frac{\widehat{\Delta}}{\sqrt{{S^2}_{obervation}}}\kern0.5em =\kern0.5em \frac{Estimate\left(\Delta {IGt}_2\right)}{\sqrt{\left({S^2}_{physician}+{S^2}_{residual}\right)}}$

For the binary item asking for “*Did participant maintain the sequences of treatment options (first standard then trials)?”,* four CG participants maintained the sequence for one of the RCT scenarios and 16 maintained the sequence for both of the scenarios (0% never, 20% once, 80% twice). For the IG, one participant did not maintain the sequence in either of the RCT scenarios, and five and 14 participants maintained the sequence in one or both RCT scenarios, respectively (5% never, 25% once, 70% twice). These differences were not observed to be significant (*p* = 0.38). Table 3 shows the mixed models results of subgroups items while Table 4 provides the results for the individual items and Additional file 1: SA contains additional descriptive data.

**Table 4:** Results of individual items of the COM-ON-rct-checklist

Item	Effect	Estimates	Standard error	Confidence interval 95%	p
Content specific items					
Explore patient’s perception	Δ IG t_2_	0.6297	0.3009	0.01998–1.2394	0.0433
Set an agenda	Δ IG t_2_	0.1507	0.2279	−0.3110-0.6125	0.5124
Introduce treatment options	Δ IG t_2_	0.1704	0.2443	−0.3241-0.6649	0.4898
Explain set up of research project	Δ IG t_2_	0.3827	0.1815	0.01531–0.7500	0.0416
Explain process of randomisation	Δ IG t_2_	0.1680	0.2285	−0.2946-0.6306	0.4666
Explain reason for randomisation	Δ IG t_2_	0.7308	0.2812	0.1615–1.3001	0.0132
Define risks and side-effects	Δ IG t_2_	−0.5135	0.2717	−1.0640-0.03703	0.0666
Define unknown effects of study	Δ IG t_2_	0.5419	0.2437	0.04846–1.0354	0.0322
Voluntariness of participation	Δ IG t_2_	0.2011	0.2473	−0.2995-0.7016	0.4212
General communication skills					
Appropriate initiation	Δ IG t_2_	0.6573	0.3743	−0.1018-1.4164	0.0876
Close discussion appropriately	Δ IG t_2_	0.04456	0.2007	−0.3618-0.4509	0.8255
Use appropriate language	Δ IG t_2_	0.2000	0.2507	−0.3075-0.7076	0.4300
Employ adequate nonverbal communication	Δ IG t_2_	−0.05739	0.1937	−0.4495-0.3347	0.7686
Take pauses	Δ IG t_2_	0.2851	0.2370	−0.1948-0.7649	0.2366
Show empathy to the patient	Δ IG t_2_	0.03647	0.2794	−0.5291-0.6020	0.8968
Encourage asking questions	Δ IG t_2_	0.1400	0.2880	−0.4430-0.7230	0.6297
Check understanding	Δ IG t_2_	0.2901	0.2546	−0.2253-0.8056	0.2616
Structure the discussion	Δ IG t_2_	0.4278	0.2245	−0.02663-0.8822	0.0643
Global					
Global evaluation	Δ IG t_2_	0.3960	0.1824	0.02671–0.7652	0.0363

Mixed model with parameter estimates, 95% confidence intervals, baseline level as covariate: CG t_1_ vs. IG t_2_

For our secondary outcome, all ten items assessing domains associated with participants’ feeling of confidence in the discussion of RCTs with the actor-patients showed significant changes, except for the item “respect information need.” This item assessed how a participant replied to differences in what and how much information a patient required, see Table 5 and Additional file 1: SB.

**Table 5:** Feeling of confidence in communication across 10 domains, calculated pre-post using a Mixed Model

Item	Effect	Estimates	Standard error	Confidence interval 95%	p	Effect sizes
Providing adequate information	Δ IG t_2_	−15.5815	5.1372	5.0889–26.0740	.005	0.5543
Ability to provide complex information about study	Δ IG t_2_	−18.9586	3.9262	11.1472–26.7700	.000	0.5356
Quality of consultation	Δ IG t_2_	−11.9280	4.1145	3.5597–20.2962	.007	0.5024
Feeling secure in consultation	Δ IG t_2_	14.4451	4.4563	−23.3206- -5.5697	.002	−0.3718
Respect of information needs	Δ IG t_2_	−6.6595	4.2640	−2.0308-15.3498	.128	0.2780
Explanation of randomization	Δ IG t_2_	−11.4534	4.0863	3.1372–19.7697	.008	0.4900
Assurance of voluntariness	Δ IG t_2_	−7.3781	3.2309	.8301–13.9260	.028	0.3767
Description of alternatives	Δ IG t_2_	−12.4441	3.9428	4.3962–20.4920	.004	0.5724
Ability to provide complex information	Δ IG t_2_	−16.8086	4.3249	8.1930–25.4242	.000	0.4488
Explanation of side effects	Δ IG t_2_	−15.5659	3.9043	7.7899–23.3420	.000	0.4573

## Discussion

In our study, an individually-tailored CST significantly improved the quality of physicians’ communication skills when discussing oncology RCTs. Post-training, the IG demonstrated significant improvement in their overall scores, as well as for a sub-group of content-specific communication skills and a global assessment of communication competency, using a standardized evaluation checklist by blinded raters. Explaining the reason for and process of randomization is a crucial part of discussing RCTs with patients. The observed improvement in the item *“explain the reason for randomization”* may, therefore, be of particular clinical significance.

Our study was the first to demonstrate the effects of an individually-tailored CST using a rigorous, randomized study design. Although the observed effect sizes were moderate, they were similar to those found in the recent meta-analysis of prior studies [25]. According to Norman and colleagues [35], a moderate effect can be interpreted as a successful outcome. In previous studies, we observed the format of our an individually-tailored CST concept was well-accepted by participants [31]. Participants’ subjective feeling of confidence also increased significantly in nine out of ten domains. Only the item, “respect information need” did not change, indicating limitations in responding to differences in patient’s information needs when discussing RCTs. Increased confidence when discussing RCTs may, in turn, result in improvements in IG participants’ communication skills. A modified training concept could be designed to address the influence of communication confidence more thoroughly. Similarly, the training could be revised to more fully address the checklist items where the observed improvement did not achieve significance (e.g., for the subgroup of general communication skill items and the binary item that assessed whether participants maintained the sequence of first discussing standard treatment options and then the trial).

### Strengths and limitations

To minimize bias in our evaluation, we randomly assigned participants to the IG or CG. Additionally, the raters were blinded as to participants’ study arm assignment. To minimize variation, we also used assessments of standardized oncology RCT discussion sessions based on real-life RCTs and patient scenarios, employing trained actor-patients. This quasi-experimental approach was helpful in focusing on one of the key elements in the accrual of clinical trials: communication skills. However, the use of actor-patients may also be viewed as a weakness, as the results demonstrated may not translate into actual practice. Further, we can only hypothesize how the observed improvement in participants’ communication skills might lead to increased recruitment rates for RCTs.

Another potential limitation was our use of a self-developed checklist [34]. Although the raters were rigorously trained to maximize internal reliability, we cannot attest to the external reliability or validity of the checklist. However, this is a common problem in communication skills research. On the one hand, Uiterhoeve [36] pointed out the need for assessment tools closely linked to the teaching content. On the other hand, this approach to assessment limits external validity.

The ICC for the global rating of communication competency was only 0.5. As a result, the significant effect observed for this rating must be viewed critically. Further, we developed the item sub-groupings theoretically. Grouping the items based on factor analysis would have been more appropriate. However, we lacked the external data set needed.

As a further limitation, different baseline scores were observed for the IG and IC. Participants with a high baseline score may have consequently faced ceiling effects. We addressed this issue by using baseline scores as covariates, thus adjusting our analysis for possible baseline differences. Despite the potential weakness and limitations of our study, the effectiveness of the tailored-CST is supported by the multiple significant, positive changes observed in the checklist and self-confidence items.

## Conclusions

Our individually-tailored CST study builds upon the prior work of others [1, 12, 30]. In the work of Brown [1, 29], a one-day CST delivered to oncologists was associated with significant improvement in three of 25 items related to the clinical and ethical information provided. These items are comparable to the content-specific communication skills item sub-group in our study. In discussing their results, the authors identified the small number of participants, the wide range of topics, and the limited training time as possible limitations. Comparatively, our CST was focused more narrowly on key topics related to discussing RCTs and utilized a longer training time. We conclude that our approach, one of being focused and individually tailored, may be more effective than an approach which covers a wide range of topics.

In the CST developed by Jenkins [12], training included eight hours of training, delivered to both research nurses and oncologists. In this second prior study, significant improvements were observed for ten out of 25 key content items. However, the participants had previous CST experience and may have been specifically predisposed to improving their communication skills. In comparison, only six of the participants in our study had prior CST experience. Our training was designed to combine training of general communication skills with content specific communication skills. Improved outcomes were observed in our study, but our CST also included a longer training time. The training of Jenkins, et al. [12] can be seen as an advanced course to be taken after general communication skills training. In comparison, the individually tailored CST evaluated in our study may be especially suited to the training of mixed groups of physicians - those without prior CST experience, as well as those with greater experience.

## Additional file

Additional file 1:
**SA.** Descriptive data of Control Group and Intervention Group results from the COM-ON-rct-Checklist by independent raters. **SB.** Descriptive data of Feeling of Confidence in 10 domains. (DOCX 22 kb)

## Abbreviations

CG: Control group
CST: Communication skills trainings
ES: Effect sizes
ICC: Interclass coefficient
IG: Intervention group
RCT: Randomized clinical trials
