# Supplementary material for: Effect of individualized communication skills training on physicians’ discussion of clinical trials in oncology: results from a randomized controlled trial
Source: BMC Cancer. 2017 Apr 13;17:264. doi: 10.1186/s12885-017-3238-0 (PMC5390387; doi:10.1186/s12885-017-3238-0)
Supplement: Additional file 1: — SA. Descriptive data of Control Group and Intervention Group results from the COM-ON-rct-Checklist by independent raters. SB. Descriptive data of Feeling of Confidence in 10 domains. (DOCX 22 kb) [file 12885_2017_3238_MOESM1_ESM.docx]

**Legends of Supplements**

**Supplement A:** Descriptive data of Control Group and Intervention Group results from the COM-ON-rct-Checklist by independent raters

**Supplement B:** Descriptive data of Feeling of Confidence in 10 domains

**Supplement A:** **Descriptive data of Control Group and Intervention Group results from the COM-ON-rct-Checklist by independent raters**

|  | **Control group (CG)** | | **Intervention Group (IG)** | |
| --- | --- | --- | --- | --- |
|  | **t_pre_**  **M (SD)**  **range*** | **t_post_**  **M (SD)**  **range** | **t_pre_**  **M (SD)**  **range** | **t_post_**  **M (SD)**  **range** |
| **Item** | | | | |
| **Content specific items** | | | | |
| Explore patient’s perception | 1.99 (0.82)  0.83-3.67 | 1.86 (1.04)  0-3.67 | 1.83 (0.73)  0.5-3.5 | 2.45 (0.95)  1-4 |
| Set an agenda | 1.78 (0.64)  0 – 3 | 1.97 (0.68)  0.67 – 3.50 | 1.43 (0.77)  0 – 3.5 | 2.07 (0.81)  0 – 3.5 |
| Introduce treatment options | 2.34 (0.96)  0.5 - 4 | 2.42 (0.72)  1.5 - 4 | 2.04 (0.92)  0.5 - 4 | 2.56 (0.85)  1 - 4 |
| Explain set up of research project | 3.2 (0.57)  2 - 4 | 2.94 (0.64)  1.5 – 4.0 | 2.90 (0.76)  1 - 4 | 3.34 (0.44)  2.5 – 4 |
| Explain process of randomization | 2.52 (0.68)  1.17 -3.5 | 2.57 (0.83)  1 - 4 | 2.28 (1.06)  0 - 4 | 2.68 (0.75)  1 – 3.67 |
| Explain reason for randomization | 1.38 (0.89)  0 - 3 | 1.67 (0.93)  0 – 3.5 | 1.46 (1.14)  0 – 3.5 | 2.42 (0.86)  0.5 - 4 |
| Define risks and side-effects | 2.39 (0.91)  0 – 3.5 | 2.81 (0.74)  1 - 4 | 2.37 (0.90)  0.5 - 4 | 2.28 (0.91)  0 – 3.5 |
| Define unknown effects of study | 2.75 (0.79)  1.5 - 4 | 2.79 (0.81)  1.5 - 4 | 2.49 (0.73)  1 -3.5 | 3.38 (0.68)  2 - 4 |
| Voluntariness of participation | 2.45 (0.86)  1- 4 | 2.77 (0.72)  1 - 4 | 2.51 (0.80)  0.5 - 4 | 2. 98 (0.86)  1 - 4 |
| **General communication skills** | | | | |
| Appropriate initiation | 2.03 (1.25)  0 - 4 | 1.83 (1.25)  0 - 4 | 1.38 (1.07)  0 -3.5 | 2.28 (1.22)  0 - 4 |
| Close discussion appropriately | 2.98 (0.84)  1.5 - 4 | 2.97 (0.74)  1 - 4 | 2.84 (0.92)  1 - 4 | 2.95 (0.83)  1 - 4 |
| Use appropriate language | 2.74 (0.74)  1.17 - 4 | 2.64 (0.85)  0 – 3.5 | 2.49 (0.80)  0.5 – 3.5 | 2.81 (0.81)  1.5 - 4 |
| Employ adequate nonverbal communication | 2.88 (0.79)  1.5 - 4 | 2.89 (0.71)  1.5 - 4 | 2.99 (0.96)  0.83 - 4 | 2.96 (0.84)  1.5 - 4 |
| Take pauses | 2.45 (0.99)  0 - 4 | 2.36 (0.75)  0.5 – 3.67 | 2.33 (0.84)  0.5 – 3.5 | 2.59 (1.03)  0 - 4 |
| Show empathy to the patient | 2.51 (1.00)  0.5 - 4 | 2.84 (0.95)  1 - 4 | 2.28 (0.93)  0.5 - 4 | 2.43 (1.13)  0 - 4 |
| Encourage asking questions | 2.08 (0.69)  1 – 3.5 | 1.89 (0.85)  0.5 – 3.33 | 1.7 (0.96)  0.5 - 4 | 1.91 (1.05)  0 - 4 |
| Check understanding | 2.1 (0.80)  1 – 3.83 | 1.95 (0.68)  1 - 3 | 1.75 (0.70)  0.67 - 3 | 2.15 (0.97)  0.5 - 4 |
| Structure the discussion | 2.23 (0.64)  1 – 3.17 | 2.38 (0.79)  0.5 – 3.83 | 1.98 (0.85)  0.33 - 4 | 2.78 (0.67)  1.5 - 4 |
| **Global** |  |  |  |  |
| Global evaluation | 2.5 (0.83)  1 - 4 | 2.57 (0.63)  1.5 – 3.67 | 2.11 (0.70)  0.83 – 3.5 | 2.85 (0.66)  1.5 - 4 |

M: = Mean; SD: = standard deviation;

*range: each physician had 2 consultations, and some consultations were rated by 2 or 3 raters. range presented here is averaged by 2 consultations and ratings. Range from 0 to 4

**Supplement B: Descriptive data of control group and intervention group pre-assessment and post-assessment- Feeling of confidence**

|  | **Control group (CG)** | | **Intervention Group (IG)** | |
| --- | --- | --- | --- | --- |
|  | **t_pre_**  **M (SD)**  **range** | **t_post_**  **M (SD)**  **range** | **t_pre_**  **M (SD)**  **range** | **t_post_**  **M (SD)**  **range** |
| **Providing adequate information** | 50.4 (20.6)  20.5 – 92.0 | 59.4 ( 17.2)  29.0 – 91.5 | 51.8 (17.2)  27.5 – 80.5 | 74.5 (15.1)  41.0 – 91.5 |
| **Ability to provide complex information about study** | 50.8 (21.0)  10.0 – 94.0 | 57.7 (17.8)  30.0 – 88.5 | 50.6 (15.1)  30.0 – 71.5 | 75.1 (10.6)  52.0 – 89.5 |
| **Quality of consultation** | 50.3 (18.9)  11.5 – 78.0 | 59.8 (14.2)  32.5 – 82.0 | 44.8 (14.6)  21.5 – 79.0 | 70.8 (13.8)  40.5 – 88.5 |
| **Feeling secure in consultation** | 50.4 (23.0)  5.5 – 88.5 | 38.7 (15.5)  14.0 – 69.5 | 46.7 (19.5)  6.0 – 78.0 | 23.7 (12.6)  4.5 – 46.5 |
| **Respect of information needs** | 59.9 (22.6)  7.5 – 95.0 | 70.6 (15.7)  43.0 – 91.5 | 56.5 (19.0)  19.5 – 84.0 | 77.2 (10.8)  59.5 – 95.0 |
| **Explanation of randomization** | 62.6 (20.9)  24.0 – 95.5 | 65.8 (16.3)  29.0 – 91.5 | 64.5 (18.6)  26.5 – 88.0 | 78.2 (13.4)  40.5 – 98.0 |
| **Assurance of voluntariness** | 70.9 (19.2)  33.5 – 97.5 | 77.8 (11.9)  55.0 – 92.5 | 68.8 (17.5)  43.5 – 94.5 | 84.8 ( 11.0)  62.0 – 99.0 |
| **Description of alternatives** | 63.4 (23.6)  15.5 – 97.5 | 65.9 (21.7)  17.5 – 98.5 | 62.0 (19.0)  22.0 – 90.5 | 77.7 (16.4)  40.5 – 99.0 |
| **Ability to provide complex information** | 49.4 (21.1)  24.0 – 91.5 | 56.0 (15.3)  35.0 – 85.0 | 52.5 (17.3)  22.0 – 77.0 | 73.3 (13.4)  50.5 – 99.0 |
| **Explanation of side effects** | 53.4 (21.6)  9.0 – 88.5 | 59.6 (19.5)  18.0 – 86.0 | 57.2 (15.4)  28.0 – 93.0 | 77.0 (11.7)  53.5 – 99.0 |

Feeling of confidence in 10 domains on a 100 mm Visual Analogue Scale (VAS) from 0 till 100.

M: = Mean; SD: = standard deviation
